# The mRNA-LNP vaccines – the good, the bad and the ugly?

**DOI:** 10.3389/fimmu.2024.1336906

**Published:** 2024-02-08

**Authors:** Botond Z. Igyártó, Zhen Qin

**Affiliations:** Department of Microbiology and Immunology, Thomas Jefferson University, Philadelphia, PA, United States

**Keywords:** mRNA vaccine, adverse event, side effect, Quo Vadis, suppression

## Abstract

The mRNA-LNP vaccine has received much attention during the COVID-19 pandemic since it served as the basis of the most widely used SARS-CoV-2 vaccines in Western countries. Based on early clinical trial data, these vaccines were deemed safe and effective for all demographics. However, the latest data raise serious concerns about the safety and effectiveness of these vaccines. Here, we review some of the safety and efficacy concerns identified to date. We also discuss the potential mechanism of observed adverse events related to the use of these vaccines and whether they can be mitigated by alterations of this vaccine mechanism approach.

## The standard and non-standard components of the mRNA-LNP COVID-19 vaccines

Regulators approving the deployment of new therapeutic or prophylactic modalities insist on strict purity criteria for the product. However, the new mRNA vaccine platform offers some novel, untested, and previously unregulated aspects of impurities during manufacture. The mRNA vaccines consist of mRNA coding for the protein-of-interest complexed with and protected by a mixture of different lipids that form nanoparticles of around 100 nm in diameter (1). The mRNA is synthesized *in vitro* using an RNA-polymerase from an *E. coli*-derived DNA plasmid serving as a template. Ideally, the plasmid and other components are eliminated during the mRNA purification step (2). However, a recent preprint study using multiple sequencing assays reported levels of DNA contamination in both Moderna and Pfizer bivalent mRNA vaccines that exceeded the levels set by the European Medicines Agency (EMA) and The United States Food and Drug Administration (FDA) (3). Whether the contaminating plasmid DNA fragments can affect human health remains to be defined. Low levels of double-stranded RNAs (dsRNA) that can form during the production process and can activate innate immune sensors and induce inflammation (4), have also been reported for both Pfizer and Moderna mRNA vaccines (5, 6). The presence of physiologically relevant levels of the dsRNA in the Pfizer COVID-19 vaccine has also been confirmed experimentally, indicating that the adaptive immune responses induced by this vaccine in mice partially depend on MDA5 protein, a dsRNA-sensor (7).

The nucleoside modifications and removal of dsRNA using HPLC were initially introduced by Karikó et al., to circumvent the activation of innate immune sensors, a characteristic of unmodified mRNAs, and the production of inflammatory cytokines, such as type I interferon, that would limit protein translation from the mRNA (4, 8, 9). The long *in vivo* half-life was desirable because the mRNA technology was initially meant to replace or deliver a therapeutic protein (10). Nevertheless, the nucleoside modification was touted as a breakthrough discovery that allowed the human use of mRNA-based vaccines (11). The importance of the inflammatory components in the mRNA-LNP platform is highlighted by the fact that highly purified mRNA (no detectable dsRNA) combined with lipid nanoparticles (LNPs) that do not contain the inflammation-inducing ionizable lipid is unable to induce innate and adaptive immune responses *in vivo*, while the LNP containing the ionizable lipid mixed with protein or mRNA supported similar adaptive immune responses (12, 13). The contaminating dsRNA in the mRNA-LNP vaccines (7), in combination with the highly inflammatory nature of the LNPs (12, 14), might essentially obviate the need for nucleoside modification from the immune sensing perspective. Thus, the critical component that transformed the mRNA into an immune response-inducing vaccine is the inflammatory LNP – initially thought to be an inert carrier/delivery vehicle for mRNA (10) – and not the nucleoside modifications.

Different levels of contaminants between vaccine lots, besides storage, transportation, and clinical handling, might explain a recent finding from Denmark that different lots of the mRNA Pfizer vaccines induced distinct levels of adverse events. Some lots caused almost no side effects, while others were associated with a medium or very high incidence of adverse events (all suspected side effects, -serious and -related deaths) (15). It would be important to determine how the same vaccine lots behaved across different countries and demographics to define whether these findings can be generalized. While contaminants likely contribute to the inflammatory nature of this platform and induction of adverse events, the LNPs’ ionizable lipid component of the mRNA-LNP vaccine is highly inflammatory (12), and as we already discussed above, it is key for the reactogenicity and immunogenicity of this platform (12, 13). Thus, hypothetically, another potential explanation for the distinct lots triggering different levels of adverse events could be that the amounts of mRNA-LNP or the mRNA : LNP ratio differed between lots (16). To assess different possibilities, it would be essential to determine the level of adaptive immune responses triggered by the different vaccine lots and if any properly stored vaccine leftovers are still available to test them for impurities and the levels of therapeutic agents. In summary, these findings highlight the need for a strict assessment of purity criteria and allowable limits for this novel vaccine class.

## Concerning assumptions made regarding the mRNA-LNP platform

Different experts and officials have made several assumption-based public statements regarding the mRNA-LNP vaccines. One of the most publicized ones was that the vaccine mRNA cannot be reverse transcribed into DNA; thus, there is no risk of insertion into the human genome (17–20). New DNA insertion into the human genome would be a serious concern if it happens on the level of stem cells of the reproductive system. In support of their statement, a modified version of Francis Crick’s central dogma of molecular biology (21) has often been cited that the information flow in eukaryotic cells is unidirectional, from DNA to RNA to protein. While the information flow in eukaryotic cells, in general, indeed is DNA to RNA to protein, in particular instances, RNA can be reverse transcribed into DNA. This process is mediated by reverse transcriptase, enzymes that are naturally associated with retroviruses. However, eukaryotic cells, including human cells, use reverse transcription-like processes to replicate telomeres and retrotransposons (22–25). With the Pfizer mRNA-LNP vaccine, it has been shown experimentally that the vaccine mRNA can be reverse-transcribed into DNA in an immortalized human hepatocyte cell line. Exposure to the mRNA-LNP vaccine also correlated with an increase in overall LINE-1 retrotransposon expression levels and localization to the nucleus (26). It has been proposed that the sequence features of the vaccines’ mRNA meet all known requirements for retroposition using LINE-1 (25). Whether these have any *in vivo* relevance remains to be determined (27). Spike protein localization to the nucleus was previously reported (28, 29). In support of studies revealing nuclear localization of the spike protein, a recent preprint study reported that the spike protein of the SARS-CoV-2, unlike other SARS viruses, contains a nuclear localization signal (NLS). The NLS allowed the transport of the spike protein to the nucleus, and it seems that the spike protein also shuttled the spike mRNA to the nucleus (30). A study also showed that SARS-CoV-2 RNA can be reverse-transcribed and integrated into the genome of cultured human cells, a process potentially mediated by LINE-1 elements, and can be expressed in patient-derived tissues. The authors propose that these findings could also possibly explain why some patients test PCR positive for SARS-CoV-2 even after clearance of the virus (31). These results, however, have been criticized as not reproducible (32, 33), infrequent and artefactual (34). While, to our knowledge, similar studies have not been performed with COVID-19 mRNA vaccines that code for full-length pre-fusion fixed form of SARS-CoV-2 spike protein, comparable transport of spike protein/mRNA to the nucleus could be expected. Because the mRNA can enter the nucleus, where it might be reverse-transcribed into DNA, this increases its potential to integrate into the genome. Furthermore, the mRNA-LNP diffuses throughout the body and can accumulate in both the testes and ovaries (5, 6) and is reported to alter the menstrual cycle in women (35, 36). Therefore, it could potentially be reaching the stem cells of the reproductive organs. These findings highlight the need to take these data and concerns seriously and conduct specific experiments to address them (25).

Another often touted feature of the vaccine mRNA is that it is degraded *in vivo* in hours or a few days, thus further limiting its potential to disrupt normal cell biology (17–20). This assumption likely arose since unmodified mRNAs have, in general, short *in vivo* half-life (37). However, human lymph node biopsies taken at different time points post-exposure to the mRNA-LNP revealed detectable levels of vaccine mRNA and spike proteins up to eight weeks (38). Circulating vaccine mRNA and spike protein have been detected in the plasma from a few weeks to several months post-vaccination (39–42). A recent post-mortem study also found vaccine mRNA in the lymph nodes of most subjects 30 days post-exposure and less frequently in their heart tissue but not in the liver and spleen (43). Thus, in light of these, we should admit to our limited understanding when it comes to how different modifications to the mRNA (5’ and 3’ modifications, the use of unique nucleotides, etc.) affect its *in vivo* half-life in the human body because no specific studies have been conducted to address this.

Modified ribonucleotides are commonly incorporated into the mRNAs of the COVID-19 vaccines to decrease their innate reactogenicity (8). However, unfortunately, their effects on mRNA translation fidelity were not defined until recently. Incorporation of N^1^-methylpseudouridine into mRNA resulted in +1 ribosomal frameshifting *in vitro* and cellular immunity in mice and humans to +1 frameshifted products from BNT162b2 vaccine mRNA translation occurred after vaccination (44). The presence of frameshifted products and associated adaptive immune responses were specific to this platform since they were not detectable with the adenovirus-based ChAdOx1 nCoV-19 vaccine (44). Whether the frameshifted products overlap with endogenous protein sequences and can contribute to developing autoimmune responses remains to be addressed.

Overall, this section highlights the danger when we assume and extrapolate in science and apply existing paradigms to new, untested platforms. Since the above issues concerning deploying this novel vaccine are particular to this platform, they must be better addressed for its future use in humans.

## How safe and effective are the mRNA-LNP vaccines?

The mRNA-LNP COVID-19 vaccines, based on early analysis of the clinical trial data, were deemed safe and effective across demographics (45, 46). However, recent peer-reviewed research studies, a wide variety of continuously increasing case reports, and publicly available adverse events databases cast doubts on the safety and effectiveness of these products.

## Safety

**Figure 1:**
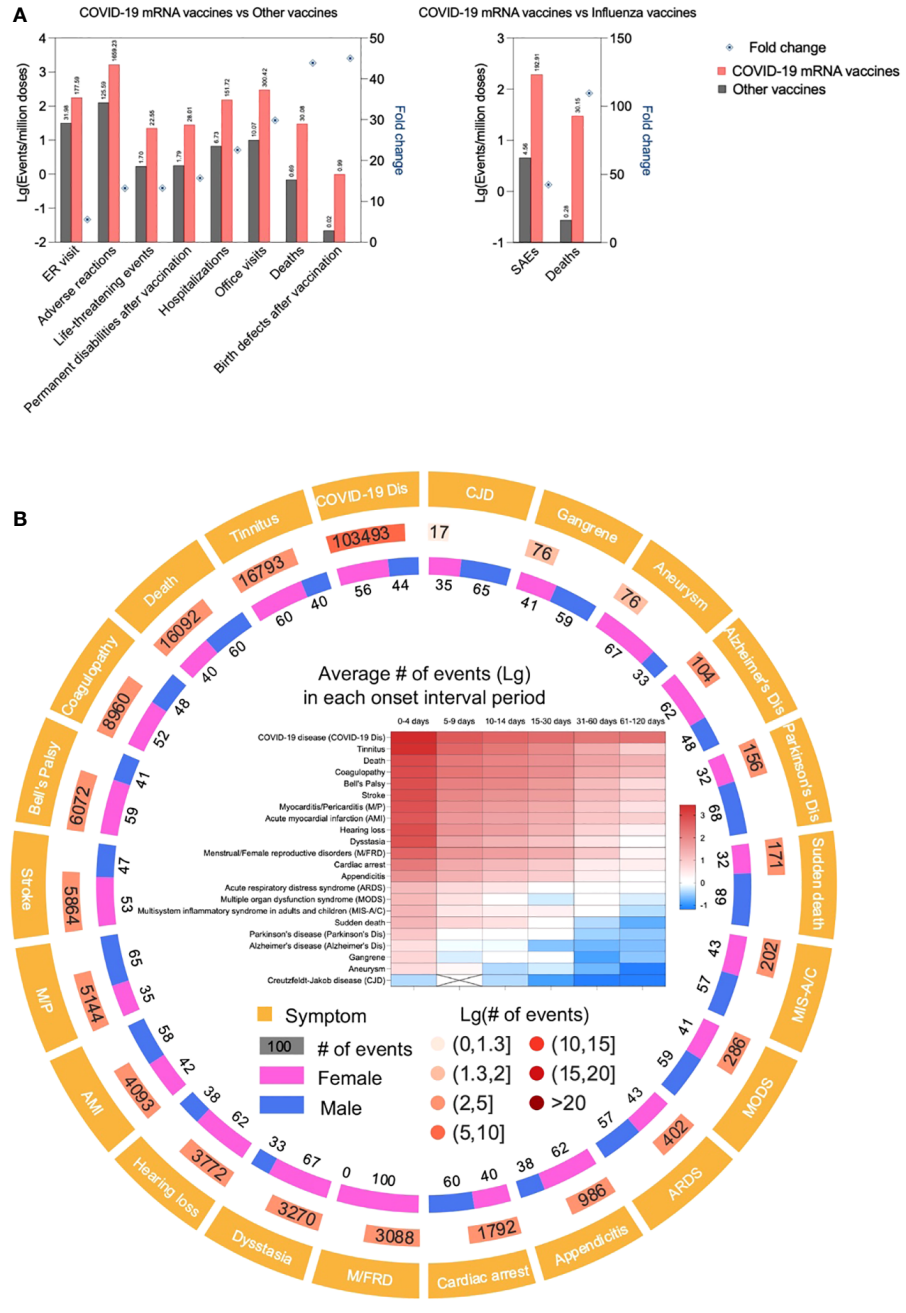
COVID-19 mRNA vaccines are associated with higher incidence of adverse events compared to other vaccines. **(A)**. Data derived from CDC VAERS and National Vaccine Injury Compensation Program (NVICP) were depicted as Lg (events/million doses) in the bar chart. The data for COVID-19 mRNA vaccine and other vaccines were from December 2020 to September 2023 and January 2006 to December 2021, respectively. The fold change was calculated as the Lg (events/million doses) ratio of COVID-19 mRNA vaccine to other vaccines or influenza vaccines.**(B)**. Adverse events of concern associated with COVID-19 mRNA vaccines. Data derived from CDC VAERS were analyzed, adverse events of concern (including AESI, adverse events of special interest, defined by CDC) were displayed as symptoms and their # of events, gender proportion and onset interval of each symptom.

Careful analysis and re-analysis of the Pfizer and Moderna clinical trial data led by Dr. Doshi, an expert in clinical trials, revealed excess risk of severe adverse events (SAEs) of special interest with both the Pfizer and Moderna COVID-19 vaccines. Combined, there was a 16% higher risk of SAEs in mRNA vaccine recipients (47, 48). This concurs with a recent admission from the German Health Minister to ~1 in 10,000 severe/permanent damage events (49). The Countermeasures Injury Compensation Program(CICP) data show that out of a total of 13,406 CICP claims ever filed, 12,854 were COVID-19 countermeasure claims, out of which 9,682 allege injuries/deaths from COVID-19 vaccines (50). Peer-reviewed case reports, including but not limited to severe inflammatory/autoimmune events of bone marrow (51–55), liver (56–58), skin (59–64), cardiovascular- (65–77), musculoskeletal- (76, 78, 79), endocrine- (80–84), and nervous system (66, 84–87), etc. that sometimes had been fatal, have been steadily increasing. However, the incidence of SAEs is hard to judge based on the number of these reports alone (88). To supplement the case report data, we performed a limited analysis of the publicly available data from the Vaccine Adverse Event Reporting System (VAERS), co-managed by the Centers for Disease Control and Prevention (CDC) and FDA (89). According to VAERS, all death reports and selected serious events of interest are investigated (89). The analyses mirror the diverse adverse effects of these therapeutics reported in the literature and might provide some information on incidence (**Figure 1**). Compared to all other non-COVID-19 vaccines combined, the incidence of adverse events is far higher for the mRNA-LNP-based COVID-19 vaccines per million doses administered (**Figure 1A**). Deaths and SAEs also often occurred soon after injection (**Figure 1B**), making it more likely to be the consequence of the vaccine and not just a random event. Nevertheless, it is essential to emphasize that one cannot establish causation simply by looking at VAERS reports. The causative relationship between the mRNA-LNP vaccine and SAEs has only been officially recognized by the regulatory agencies for peri- and myocarditis affecting primarily young males (90). While most symptomatic patients might be young males, recent, *in vivo* physiological tests, such as heart glucose uptake, showed a 40% increase in asymptomatic vaccinated patients irrespective of gender and demographics (91), potentially suggesting a much broader impact. These findings are also supported by a postmortem study, in some of which an autopsy revealed heart inflammation and the presence of vaccine RNA (43). Whether the increase in heart attacks and death in young people (92, 93) might be linked to these vaccines remains to be determined.

## Efficacy

The effectiveness of these therapeutics in preventing infections and limiting the spreading of the virus has been highly eroded from the early reports (94), and nowadays, their efficacy is mainly limited to potentially decreasing the disease severity and death in susceptible people (95). Excess inflammation caused by an overreacting immune system (cytokine storm) is one of the major pathological features in patients with severe COVID-19 (96). Thus, hypothetically, if exposure to the mRNA-LNP vaccine leads to a dampened systemic inflammatory response, that may explain why vaccination also reduced disease severity in the case of the delta and omicron variants, in which case the antibodies induced by the original vaccines were not (97), or minimally neutralizing (98). In support of this hypothesis, Dr. Netea’s group reported dampened transcriptional reactivity of the immune cells and decreased type I interferon responses in vaccinated individuals to secondary viral stimulation (97), while our group described inhibition of adaptive immune responses and alteration in innate immune fitness in mice with this platform (99). The immune-tolerant environment induced by these vaccines is further supported by recent studies that have discovered a correlation between an increased number of prior mRNA vaccine doses and a higher risk of catching COVID-19 (100–102). Thus, these data suggest that these vaccines’ efficacy in decreasing disease severity and death might lie with their previously undiscovered immune suppressive characteristics. These findings further highlight the need for rigorous pre-clinical studies to limit potential unexpected consequences for novel platforms.

## mRNA-LNP-associated adverse events – both inflammatory and inhibitory

The SAEs reported with the mRNA-LNP platform are very diverse. The SAEs are likely caused by the combination of the mRNA-LNP vaccine components (103) and potentially by direct toxicity and biological action of the spike protein itself (104–108). Here, we will focus on SAEs likely mediated by the immune system. The SAEs can be divided into inflammatory and anergic/inhibitory categories from an immunological perspective. Inflammatory side effects include acute reactogenicities (fever, headache, fatigue, myalgia and arthralgia, chills, etc.) (45, 46), inflammatory/autoimmune, anti-PEG mediated CARPA (109), and other related events that involve activation of the innate and adaptive immune systems. Anergic side effects, i.e., immune suppression, are presented as virus reactivation where viruses like varicella-zoster virus (VZV) (110, 111) and hepatitis C virus (112) reoccur following COVID-19 mRNA vaccine injection. Furthermore, have also been reported to likely increase the risks in sensitivity to infections (100, 101, 113) and potentially disturbance of cancer immunosurveillance (114–117). How can a platform both activate and suppress immune responses?

## Inflammatory responses

As discussed above, the inflammatory nature of this therapeutic is linked to the ionizable lipid component of the LNPs (12), which, in effect, might further be accentuated by potential pro-inflammatory contaminants, such as dsRNA (7). The acute reactogenicity responses observed with these therapeutics, such as fever, headache, fatigue, myalgia, arthralgia, chills, etc., are likely triggered by the release of a variety of high amounts of innate inflammatory cytokines, such as IL-1β, IL-6, GM-CSF and type I interferon (7, 12) upon exposure. This inflammatory environment induced by this therapeutic, and that the mRNA-LNP has additive effects with other inflammatory agents, such as LPS (118), could potentially support flareups with pre-existing autoimmune conditions and/or create conditions for novel autoimmune responses to develop in susceptible people. Since the mRNA-LNP diffuses throughout the body (6, 10, 41, 42), and LNPs can deliver mRNA into any cell type and enable its translation, it is reasonable to consider an off-target translation of protein-of-interest in non-APCs, which then could be under attack by the antigen-specific adaptive immune responses (103). Therefore, the destruction of spike protein- or frameshifted-protein-expressing cells by the immune system throughout the body might be responsible for some of the SAEs reported with this platform, such as COVID arm, peri/myocarditis, and inflammatory responses affecting the brain, liver, bone marrow, etc.

## Tolerogenic responses

The observation that protection against omicron infection waned gradually by month after the booster and seventh month and thereafter, the incidence of infection was higher among people who had the booster compared to those with only the primary series (119), and COVID-19 incidence increases with the number of shots (100–102) and immunoreactivity decreases (97), and viruses can reactivate (110, 112), and cancer patients relapse or new cancer develop (114–117, 120), seems to indicate that exposure to the mRNA-LNP platform puts people or certain people into a semi-immunodeficient/immunosuppressed state. In line with this, mouse studies have shown that even one-time exposure to mRNA-LNP or LNP can inhibit adaptive immune responses and alter innate immune fitness in an inheritable fashion (99). While one-time exposure decreased susceptibility to subsequent influenza infection, the mice became significantly less resistant to systemic yeast infection. Whether the increase in sepsis- (121), nosocomial- (113, 122), and certain fungal infections (123–126) incidence are related to the vast exposure to the mRNA-LNP platform or COVID-19 and or other pandemic-related measures remains to be addressed.

The ionizable lipids of the LNPs are synthetic and are estimated to have a 20-30 day *in vivo* half-life (5). Thus, exposure to these vaccines may lead to an early high level of inflammation followed by a long-lasting low level of chronic inflammation. Chronic inflammation can lead to non-responsiveness and anergy of the immune system (127), thus potentially contributing to some of the viral reactivation and increased susceptibility to infections reported in association with this platform. Furthermore, as presented above, evidence suggests that contrary to expectations, spike mRNA can also remain intact for months following mRNA vaccination, allowing long-term spike protein expression. This correlates with the presence of germinal centers that last for months. Continuous stimulation with antigens can lead to aberrant T and B cell responses. Continuous antigen exposure might promote the isotype switch to IgG4 recently observed in roughly half of the individuals receiving three doses of an mRNA vaccine but not by adenovirus vaccines (128, 129). Prior exposure to natural SARS-CoV-2 infection prevented the mRNA vaccines from inducing IgG4 switching (130), indicating that the initial priming of the immune system imprints and determines the subsequent humoral immunity. IgG4 is generally considered anti-inflammatory and known to poorly facilitate opsonization, complement fixation, and antibody-dependent cellular toxicity. Still, it can also be pathogenic in the case of the autoimmune disease pemphigus vulgaris and some forms of myasthenia gravis (131). IgG4-related disease also appears to have a higher risk of overall cancer (132). The induction of IgG4-producing B cells relies on help from a subset of T follicular helper (Tfh) cells characterized by the production of high amounts of IL-10 (133). These IL-10-producing Tfh cells are generated in the presence of continuous antigen stimulation and were first identified in chronic viral infection (133), and were later shown to be present in IgG4-related human diseases (134). Whether the mRNA-LNP platform induces this unique type of Tfh cells and the *in vivo* relevance of the observed switching to IgG4 isotype with the mRNA-LNP platform remain to be determined (135).

**Figure 2:**
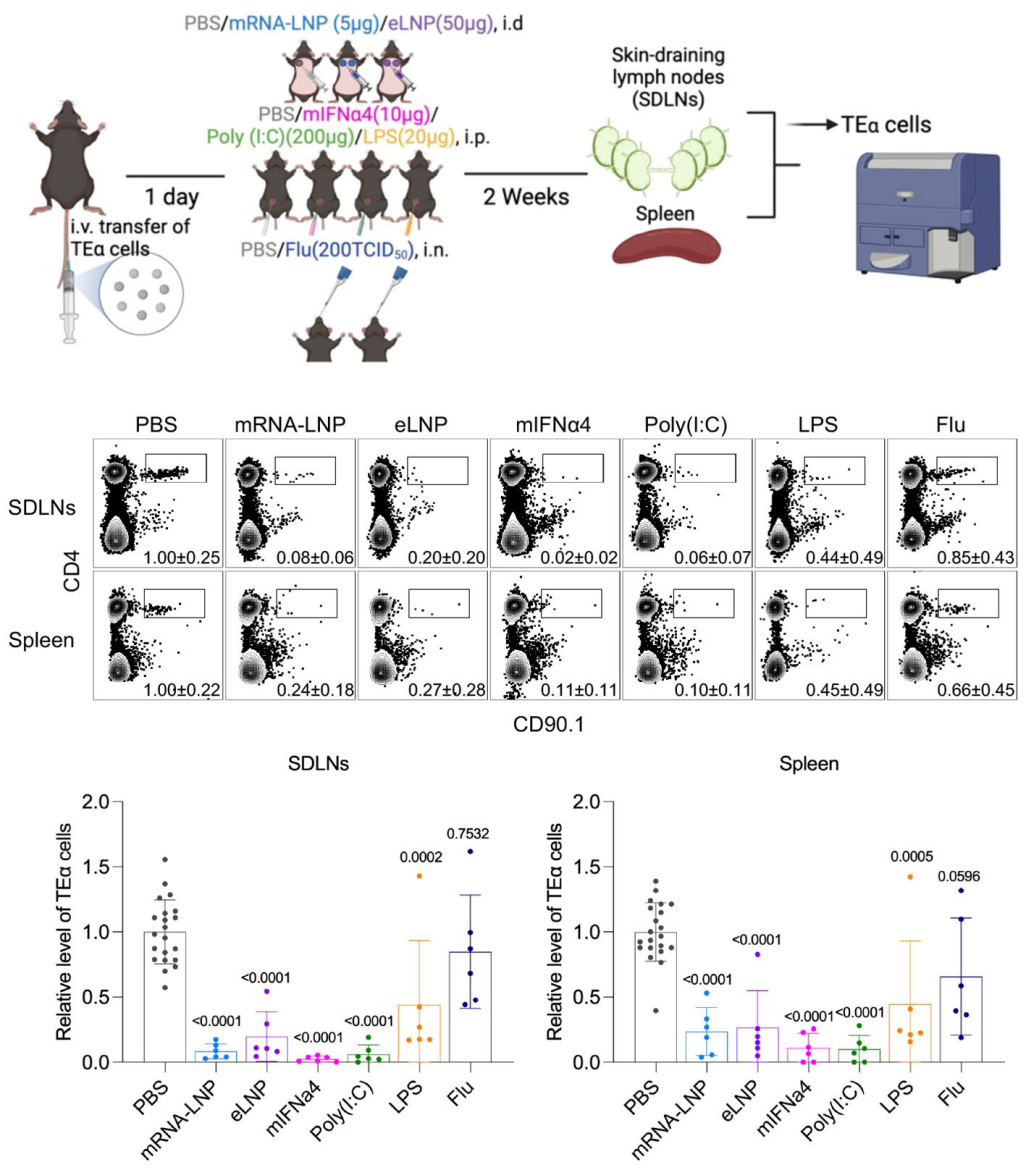
Exposure to mRNA-LNP and other type I IFN-producing reagents potentially induce out-of-sequence stimulation. Experimental design. Mice were adoptively transferred with TEα cells through tail vein one day before exposure to different types of inflammatory reagents at indicated routes. Two weeks later, draining and non-draining lymph nodes and spleens were harvested for detection of TEα cells by flow cytometry. Representative flow plots showing TEα cells (CD4+CD90.1+, gated from live CD3+ population) from different organs under each treatment condition, and corresponding summarized bar chart (bottom). Data were pooled from at least two independent experiments. Each dot stands for one mouse. The relative level of TEα cells was normalized to PBS group. Comparisons between PBS group and treatment groups were made by One-way ANOVA test.

Our laboratory has recently proposed another potential complementary mechanism to immune suppression (99). Activation and differentiation of T cells into effector cells are thought to rely on three signals received from antigen-presenting cells (APCs), such as dendritic cells (DCs). The first signal is in the form of peptide-MHC, the second signal is membrane-bound co-stimulation, and the third signal is in the form of soluble cytokines. If the T cells receive the third signal before the first one, it is called out-of-sequence stimulation, which can lead to the T cells’ death or render them anergic and non-responsive (136, 137). Exposure to mRNA-LNP or LNP leads to a rapid release of inflammatory cytokines (12), including type I interferons (7) that are known to induce out-of-sequence stimulation, which likely exposes the adaptive immune cells, including the T cells, to an inflammatory environment before the mRNA is translated into protein and presented on DCs. Thus, with a high likelihood, one of the potential flaws of the mRNA-LNP platform, unlike the old-school vaccines where the antigen presentation and inflammation coincide, is that it exposes the adaptive immune cells to an out-of-sequence stimulation, which might worsen upon further exposure. To bring experimental evidence that out-of-sequence stimulation might exist with the mRNA-LNP platform, we transferred naïve congenically marked Eα-specific CD4 T cells into WT B6 mice, and then we exposed the mice to mRNA-LNP or LNP or PBS. Two weeks later, some of the mice were left untreated while others were immunized with Eα antigen to trigger the proliferation of the transferred T cells. Four days later, the analysis of the skin-draining lymph nodes revealed a roughly tenfold decrease in the adoptively transferred T cell numbers in animals exposed to mRNA-LNP or LNP, irrespective of their immunization status (99). Thus, these data suggest that the inflammatory environment created by the LNPs is detrimental to naïve T cells. In another set of experiments, we compared the effect of mRNA-LNP and LNP to IFNα, or factors that induce inflammation or influenza infection, on the survival of adoptively transferred naïve CD4 T cells. Similar to mRNA-LNP and LNP, we found a decrease in T cell precursor numbers if we treated the mice with type I interferon (IFNα and poly (I:C) (triggers type I interferon secretion) (**Figure 2**). The high dose of LPS led to an intermediate phenotype, while sub-lethal influenza exposure had no significant effect on the adoptively transferred CD4 T cells (**Figure 2**). The effect was not limited to TEα cells since OT-II CD4 T cells responded similarly (data not shown). Whether endogenous T cells are similarly affected remains to be determined. Notably, the observed changes were likely systemic since the direct draining organs, the skin-draining lymph nodes, and the distant spleen showed similar trends. These data align with our previous findings that exposure to mRNA-LNP or LNP leads to systemic inhibition of adaptive immune responses (99). All the treatments used, except exposure to influenza, led to a significant decrease in the transferred naïve T cell numbers. The absence of an effect with influenza was not due to a lack of infection since mice exposed to the virus lost significant weight (data not shown). The LPS exposure, while at a lower degree, also reduced the naïve T cell numbers. Whether the effect in this case was mediated by other cytokines than type I interferon or by indirect induction of type I interferon, or the effect might be limited to the high dose of LPS that we used, remains to be determined. Overall, these data support the potential existence of a type I interferon-mediated out-of-sequence stimulation with the mRNA-LNP platform. However, further studies will be needed to identify the inhibition’s mechanistic details and determine whether these data are translatable to humans.

## Quo Vadis mRNA-LNP?

Several severe side effects have been reported with the mRNA-LNP platform, which, to preserve human health, should be carefully investigated and addressed before further use. We think that making the ionizable lipid biodegradable, thus decreasing its *in vivo* half-life, could potentially solve or limit the presence of chronic inflammation, which is detrimental to adaptive immune responses. Along the same line, the half-life of the mRNA should also be carefully adjusted to prevent chronic antigen stimulation of T and B cells. Whether the ionizable lipid component of the LNPs, similar to LPS (138, 139), can promote innate tolerance upon multiple exposures must be defined. When designing vaccines, especially booster shots targeting potential new variants, basic immunology on how antigen dose, pre-existing antibodies, and original antigenic sin (140) might affect the outcome should be considered. The out-of-sequence stimulation with the mRNA-LNP platform could be decreased using an “intelligent” design that would trigger inflammation only after the antigen has been translated from mRNA and it is ready to be presented on the surface of APCs. The present formulation of LNP makes them accumulate in specific organs more than in others, a characteristic that will persist regardless of the RNA cargo. Thus, to decrease the chance of inducing autoimmune responses and killing self-cells, the mRNA-LNP should be targeted to DCs to limit the expression of antigens to only APCs (103). The generation of frameshifted products due to the use of specific non-standard nucleotides should be remedied as proposed (44). To put people’s concerns to rest regarding the safety of the mRNA, studies should be conducted in NHPs and humans on the mRNAs’ *in vivo* half-life and whether there is any *in vivo* transcription into DNA and potential genomic insertion. Finally, since real-life data revealed potential severe gaps in quality control, stricter oversight of pharmaceutical companies from independent health officials is desirable and should be implemented without delay.

## Brain bites

Several fundamental questions persist surrounding the pandemic measures and the adoption of this new vaccine platform. Rather than advocating for retraction and censorship (141), fostering open dialogue, considering all perspectives, and employing logic and reasoning for better future preparedness is crucial. While some, despite the concerns presented above or discussed elsewhere (142, 143), would likely argue that the measures were justifiable, citing lives saved, the robustness of supporting data raises important inquiries. Estimates relying on mathematical modeling (144) necessitate scrutiny, particularly concerning the quality of input data. For instance, reliance on official death toll statistics and not accounting for demographic variations in the virus infection fatality rate (IFR; the likelihood of death if you become infected) prompt questions about the accuracy of reported figures. Why did official statistics include both individuals who died from and with COVID-19? Why does the CDC estimate the flu-associated hospitalization and death for the adult population, arguing that it cannot be accurately determined (145) but can give us exact numbers for COVID-19? Does the IFR data (146, 147) support the official death toll numbers?

The unprecedented mass vaccination with a vaccine that minimally protects from getting infected and spreading the virus (94) during a pandemic prompts critical reflections: Was it a sound strategy? Was herd immunity a realistic expectation? Did this strategy inadvertently accelerate virus mutations, and could a more targeted focus on vulnerable populations have yielded better results? (148–156) The decision to opt for the mRNA-LNP platform over traditional methods requires scrutiny, considering both its advantages (speed of production, ease of updating) and limitations (patents, production constraints, affordability, unknown short- and long-term side effects). Why focus on a single virus protein with a high mutation rate? Was the vaccines’ ease of updating feature critical to fighting against variants? Why was basic immunology knowledge on how antigen dose, repeated boosters, pre-existing antibodies, antigenic sin, etc., affect the immune system mostly ignored during the pandemic?

Amidst a shared pool of scientific knowledge, divergent decisions by countries like Sweden, which chose not to recommend vaccinations for specific age groups (157), kept schools open and reported that no child with COVID-19 died (158, 159), underscore the need for a deeper understanding of varied perspectives. Why censor the “Great Barrington Declaration,” (160) which advocated a model similar to Sweden’s, emphasizing the protection of the vulnerable? (161) This ongoing list of inquiries highlights the nuanced complexity of these issues, urging a thorough examination.

## Going forward

Numerous discussions have centered around the impact of influential scientists who advocate for drastic measures to address vaccine hesitancy and public trust erosion, potentially compromising academic freedom through censorship and intimidation. However, during the pandemic, actions like silencing dissenting voices, coupled with policy decisions often reliant on assumptions rather than robust experimental data, may have inadvertently undermined both science and public confidence. To rebuild trust, it is crucial to return to the fundamental principles of scientific inquiry. Scientists should embrace their training and be committed to questioning every assertion, regardless of the source. This approach guards against groupthink and herd mentality. Rigorous analysis of all available data using critical thinking and reasoned judgment, unaffected by conflict of interests, is essential to formulate a well-rounded perspective. Communicating transparently and honestly with society is equally vital. Acknowledging the inherent uncertainties in biology, our representatives should convey both what is known and what remains uncertain. Science is inherently dynamic, perpetually evolving as new knowledge emerges. It is imperative to emphasize that nothing in biology is absolute, and pursuing knowledge demands ongoing questioning and exploration. By adhering to these principles, we can foster a renewed trust in the scientific process and its capacity for growth and refinement.

## Data availability statement

The original contributions presented in the study are included in the article/supplementary material. Further inquiries can be directed to the corresponding author.

## Ethics statement

The animal study was approved by Institutional Care and Use Committee at Thomas Jefferson University. The study was conducted in accordance with the local legislation and institutional requirements.

## Author contributions

BI: Conceptualization, Investigation, Supervision, Writing – original draft, Writing – review & editing. ZQ: Data curation, Formal Analysis, Investigation, Methodology, Visualization, Writing – review & editing.
